# Excision of Hemorrhoids

**Published:** 1901-04

**Authors:** William L. Dickinson

**Affiliations:** Saginaw, Mich., Professor of rectal diseases, Saginaw Valley Medical College


### EXCISION OF HEMORRHOIDS.

By WILLIAM L. DICKINSON, M. D., Saginaw, Mich.,
Professor of rectal diseases, Saginaw Valley Medical College.

HEMORRHOIDS, either internal or external, can be successfully removed by excision, and in most cases with but little pain following their removal. In this brief paper I will endeavor to give my method of operating and also the after-treatment required.

External hemorrhoids should be made as nearly aseptic as possible by the usual means, and then injected with a 5 per cent. solution of nirvanine. Now grasping with forceps, we lift the hemorrhoid well up, and with scissors curved on the flat, and cut the tumor off close to its base. Curette the base, using a sharp instrument, in order to remove every vestige of the hemorrhoid. The hemorrhage is usually slight and easily controlled by pressure with a gauze compress wrung out of hot water and applied for two or three minutes. If the compress does not control the hemorrhage, we put on a hemostatic forceps for a few minutes. When the hemorrhage has stopped, we unite the two sides of the incision with fine catgut, and apply a sterilised gauze pad held in place by a T bandage. It is well to keep the patient in the house and in a recumbent position for four to six days, when the parts will be healed.

In the treatment of internal hemorrhoids by excision, we must prepare our patient for the operation by giving three improved compound cathartic pills two nights before the day of the operation; the next day give half an ounce of sulphate of magnesia in six ounces of hot water every four hours until four doses are given.

The morning of the operation an enema of plain sterilised hot water is to be given, and now we have the bowels so free of fecal matter that it will not be necessary to have them moved for five or six days following the operation. We are now ready for the operation. The patient is placed on the operating table and the anesthetic given. The anal region is scrubbed with green soap, and washed with corrosive sublimate solution, and then with alcohol.

The sphincters are now dilated until all resistance is overcome; then we apply five or six of Pratt's T forceps, handing them to our two assistants, who now pull down and outward upon them, thus exposing to view the mucous membrane of the bowel for a distance of an inch or an inch and a half. Each internal hemorrhoid in turn is grasped with a forceps, elevated as much as possible, and cut off with the scissors. Curette the base of each hemorrhoid and stop the hemorrhage with a compress wrung out of hot water. We now dust the parts with orthoform, remove the T forceps and pack the lower end of the rectum with a strip of iodoform gauze one inch wide, that has been lubricated with sterilised vaseline. It is better to use the vaseline on the gauze, as it makes it easier to remove it when necessary.

Hemorrhoids treated in this way are not very painful and it only confines the patient to the bed about one week. We use the pad and T bandage after operation upon internal hemorrhoids, as it makes it much more comfortable for the patient.

The advantages of this operation are less pain and shorter time confined to the bed and house. I have frequently been asked the question as to the danger of secondary hemorrhage, and will say that I have not had a case of secondary hemorrhage and have operated on a number of patients by this method. If we are careful to pack the lower end of the rectum tightly with iodoform gauze, there cannot be any hemorrhage immediately following the operation, and after twenty-four or forty-eight hours, when the gauze is removed, in my judgment there is no longer any danger of it.

917 Genesee Avenue.

---

Alabama has declared wisely through a recent statute enacted by her legislature, that christian scientists and faith-curists cannot longer practise their medical legerdemain in that state.

